# Anticancer activity of sodium propionate in hepatocellular carcinoma cells via downregulation of *HDAC2* and *HDAC6*

**DOI:** 10.1007/s10822-026-00967-3

**Published:** 2026-11-06

**Authors:** Ganapati Pakkirappa Yadav, Pradeepa Krishnappa, Kumaraswamy H M, Vinay C Sangamesh, Pavan Gollapalli, Sapna K, Ravindranath S Bilachi

**Affiliations:** 1 Department of Biotechnology, Sahyadri Science College, Kuvempu University, Shivamogga, Karnataka 577 203 India; 2 Department of Biotechnology, Jnana Sahyadri, Kuvempu University, Shankaraghatta, Karnataka 577 451 India; 3 NITTE (Deemed to be University), Nitte University Center for Science Education and Research, Deralakatte, Mangalore, Karnataka 575018 India; 4 Department of Bioinformatics and Biostatistics, NITTE (Deemed to be University), Nitte University Center for Science Education and Research (NUCSER), Panner campus, Deralakatte, Mangalore, Karnataka 575018 India; 5 Manipal Institute of Technology, Manipal Academy of Higher Education, Manipal, India

**Keywords:** ADMET profiling, Epigenetic modulation, Hepatocellular carcinoma, Histone deacetylases, Molecular docking, Sodium propionate

## Abstract

Hepatocellular carcinoma (HCC) is an aggressive malignancy driven by epigenetic dysregulation. This study investigates the anticancer potential of sodium propionate (SP) as an epigenetic modulator targeting histone deacetylases *(HDACs)* using an integrated in silico and in vitro approach. Bioinformatics analyses were conducted using publicly available HCC transcriptomic datasets from GEO database, disease gene database (GeneCards database), and chemical–gene interaction networks (Comparative Toxicogenomic Database) to identify potential therapeutic targets, *HDAC2* and *HDAC6* as key therapeutic targets in HCC. Computational ADMET screening indicated SP possesses favourable drug-likeness, high oral bioavailability, and low toxicity. Molecular docking and 500-ns molecular dynamics simulations showed stable SP binding to *HDAC2*, with ligand RMSD of 1.5-2.0 Å, protein RMSD of 3.0-4.5 Å, and active-site RMSF < 1.0 Å, supported by persistent Zn²⁺ coordination and hydrophobic interactions. In vitro evaluations in HepG2 cells revealed distinct dose-dependent cytotoxicity (IC₅₀ = 8.2 mM) and pronounced morphological alterations. Furthermore, SP treatment strongly downregulated the mRNA expression of *HDAC2* (0.31-fold, *p* < 0.001) and *HDAC6* (0.60-fold, *p* < 0.05). This suppression was accompanied by a significant, dose-dependent increase in caspase-3/7 activity, confirming apoptotic induction. These findings suggest SP functions as a dual-action epigenetic modulator in HCC, promoting apoptosis by downregulating *HDAC2/6* expression and potentially inhibiting their enzymatic activity. However, biochemical assays, protein-level validation, and in vivo HCC models are necessary to validate this inhibition.

**Graphical Abstract:**

**Figure:**
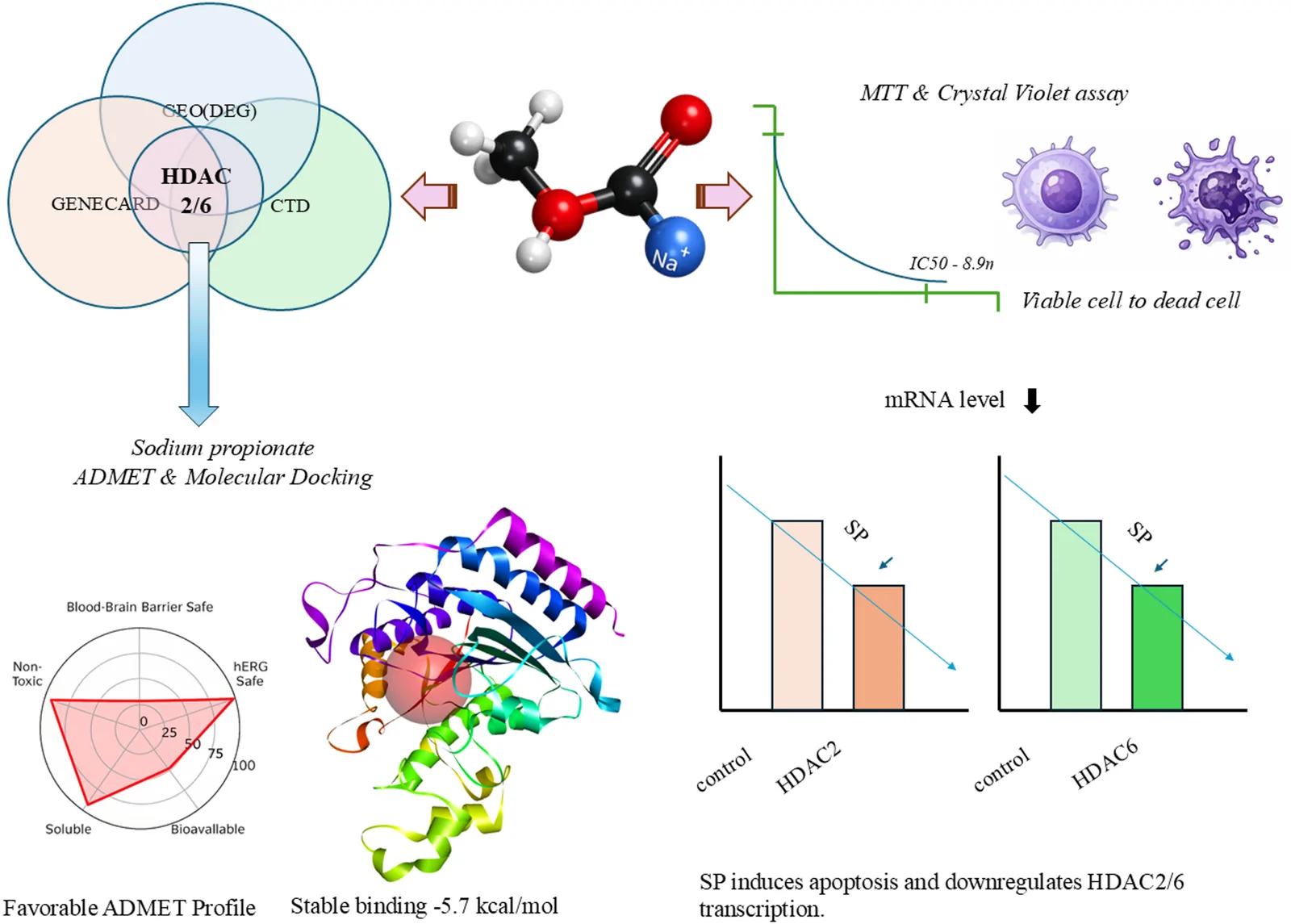

## Introduction

Hepatocellular carcinoma (HCC) represents a formidable global health challenge, currently ranking as the sixth most frequently diagnosed malignancy and the third leading cause of cancer-related mortality worldwide [1]. The epidemiological landscape of HCC is rapidly shifting, while viral hepatitis has historically been the primary driver, metabolic dysfunction-associated steatotic liver disease (MASLD) is emerging as a dominant aetiology, fuelled by the global rise in obesity and metabolic syndrome [2]. Despite advancements in surgical resection, liver transplantation, and systemic therapies such as sorafenib and immune checkpoint inhibitors, the prognosis for advanced HCC remains dismal. High rates of recurrence and drug resistance keep the 5-year survival rate below 20% [3, 4]. This persistent clinical failure underscores the urgent need to identify novel therapeutic targets and agents that can dismantle the complex molecular machinery driving hepatocarcinogenesis.

Epigenetic dysregulation, characterized by aberrant chromatin remodeling, is a central feature of hepatocellular carcinoma, with histone deacetylases (HDACs) frequently overexpressed in HCC and contributing to transcriptional silencing of tumor suppressors and activation of oncogenic pathways [5]. Among HDAC family members, *HDAC2* (Class I) and *HDAC6* (Class IIb) have emerged as promising druggable targets. *HDAC2* is predominantly nuclear and upregulated in HCC, where it promotes cell-cycle progression and autophagy-mediated survival by repressing negative regulators such as p21 and PTEN [6]. By contrast, *HDAC6* is largely cytoplasmic and regulates cytoskeletal dynamics, with its overexpression enhancing metastatic potential and invasiveness through the deacetylation of α-tubulin and cortactin [7, 8].

Short-chain fatty acids (SCFAs), produced through gut microbial fermentation of dietary fiber, have emerged as bioactive metabolites that modulate host metabolism, immune function, and gene regulation [9]. Through the gut–liver axis, these metabolites are directly delivered to the liver via the portal circulation, enabling them to exert pronounced physiological and molecular effects [2]. Sodium propionate (SP), a three-carbon SCFA, is of particular interest because of its ability to inhibit cancer cell migration, induce apoptosis, and remodel the tumor microenvironment in breast and colorectal cancer [10, 11]. However, despite the liver being a primary target organ for gut-derived SCFAs, the transcriptional basis of SP-mediated epigenetic regulation in hepatocellular carcinoma remains insufficiently understood. Defining this mechanism is particularly important, as transcriptional repression of oncogenic histone deacetylases may provide sustained anticancer activity with reduced toxicity compared to conventional synthetic *HDAC* inhibitors. In this context, the present study evaluates the anticancer potential of SP in hepatocellular carcinoma, focusing on its ability to suppress HCC progression through transcriptional downregulation of key *HDAC* isoforms. By integrating complementary in silico and in vitro approaches, this study provides a clear mechanistic framework supporting SP as a metabolically derived epigenetic modulator with therapeutic relevance in HCC.

## Materials and methods

### In silico study

#### Data sources and gene expression analysis

To construct a robust foundation for target selection, a multi-omics approach was employed. Gene expression datasets relevant to hepatocellular carcinoma were systematically retrieved from the Gene Expression Omnibus (GEO) database (https://www.ncbi.nlm.nih.gov/geo/). The selected datasets (GSE14520, GSE76427, GSE202853, GSE112790, GSE104580, and GSE121248) encompass a broad spectrum of clinical stages and etiologies, ensuring that the identified targets are universally relevant to HCC pathogenesis. Each dataset was processed using the GEO2R interactive web tool to identify Differentially Expressed Genes (DEGs) between tumor and non-tumor liver tissues. The selection criteria were stringent, with statistical significance defined as an adjusted p-value < 0.05 and a magnitude of change defined by a |log2 fold change| > 1.0. This generated a pool of 4,673 biologically significant DEGs.

Simultaneously, a knowledge-based dataset was curated from the GeneCards database (https://www.genecards.org) using the query “hepatocellular carcinoma,” yielding 9,891 genes with documented associations to liver cancer. To bridge the gap between disease pathology and chemical intervention, the Comparative Toxicogenomic Database (CTD) (https://ctdbase.org) was queried for genes known to interact with “Sodium Propionate.” This chemical-gene interaction search yielded a focused set of 24 genes. The intersection of these three distinct datasets (DEGs, Disease-Associated, and Chemical-Interactive) was visualized using a Venn diagram (https://www.interactivenn.net/), identifying a core signature of overlapping genes *HDAC2* and *HDAC6* that served as high-priority candidates for subsequent analysis.

#### ADMET and toxicity prediction

The pharmacokinetic behaviour and safety profile of SP were evaluated using the ADMET-AI platform (https://admet.ai.greenstonebio.com/) a state-of-the-art machine learning tool designed for the evaluation of chemical libraries. The SMILES notation for SP was utilized to predict a comprehensive array of parameters: Absorption: Human Intestinal Absorption (HIA), Caco-2 permeability, and P-glycoprotein substrate specificity. Distribution: Blood-Brain Barrier (BBB) penetration and Fraction Unbound (Fu). Metabolism: Inhibition or substrate potential for major Cytochrome P450 isoforms (CYP1A2, CYP2C9, CYP2C19, CYP2D6, CYP3A4). Toxicity: Assessment of acute oral toxicity (LD50), hepatotoxicity (drug-induced liver injury—DILI), cardiotoxicity (hERG channel inhibition), mutagenicity (AMES test), and carcinogenicity. This profiling confirms the potential of SP as a therapeutic agent, supported by its established role as a short-chain fatty acid (SCFA) capable of modulating epigenetic regulators such as the acetyltransferase p300 [12]. Such comprehensive screening ensures that SP could be safely repurposed as a drug, even for patients with compromised hepatic function.

#### Molecular docking studies

The crystal structures of *HDAC6* (PDB ID: 5EDU) and *HDAC2* (PDB ID: 3MAX) were obtained from the Protein Data Bank (PDB; https://www.rcsb.org) and prepared using Maestro (version 14.1.138) within the Schrödinger Suite (Release 2024-3). Protein preparation was performed using the automated Protein Preparation Workflow [13], which included the addition of missing atoms, assignment of correct bond orders, optimization of the hydrogen-bonding network, and protonation at physiological pH (7.0). Missing loops and side chains were reconstructed using the PRIME module [14], while crystallographic water molecules and co-crystallized ligands were removed. The prepared protein structures were subsequently energy-minimized using the OPLS3e force field [15] with an RMSD convergence threshold of 0.3 Å, yielding geometrically and energetically optimized models suitable for molecular docking studies. Receptor grids were generated around the Zn²⁺ ion using the Receptor Grid Generation module to define the active sites. The three-dimensional (3D) structure of SP was acquired in Structure Data File (SDF) format from the PubChem database (https://pubchem.ncbi.nlm.nih.gov/) (CID: 137663). Ligand was prepared with the LigPrep module [16] to generate energetically favourable 3D conformation. Docking was performed using the Glide Ligand Docking module in extra precision (XP) mode [17]. Prior to evaluating SP, the docking protocol was validated by re-docking the native co-crystallized ligands—N-(2-aminophenyl) benzamide for *HDAC2* and trichostatin A for *HDAC6*—into their respective Zn²⁺-centered grids. The optimal docked poses superimposed onto their native experimental structures with an RMSD of ≤ 1.0 Å, confirming the reliability of the docking parameters. The docked poses of SP were analyzed based on their docking scores and key binding interactions within the active sites of *HDAC6* and *HDAC2*, and were subsequently selected for molecular dynamics simulations to further evaluate their stability and interaction profiles [18].

#### Molecular dynamics simulations

Molecular dynamics simulations were performed using Desmond [19] in the Schrödinger Suite (2024). The HDAC2 complex was selected for dynamic analysis because it showed a more favourable docking score and post-docking interaction profile than HDAC6. The system was prepared using the OPLS4 force field [20] and solvated with the TIP3P water model [21, 22] in a cubic box. The system was neutralized with 0.15 M Na⁺ and Cl⁻ ions to approximate physiological conditions. Simulations were run for 500 ns under an NPT ensemble at 300 K and 1.01325 bar [23]. Temperature was regulated using the Nosé–Hoover thermostat with a relaxation time of 1.0 ps [24]. Pressure was controlled using the Martyna–Tobias–Klein (MTK) barostat with a relaxation time of 2.0 ps and isotropic pressure coupling [25]. Long-range electrostatic interactions were treated using the U-series method [26], while the short-range interaction cutoff was set to 9.0 Å [27]. These parameters define the thermodynamic conditions and nonbonded interactions during the simulation. The Simulation Interaction Diagram module was used to analyse protein–ligand interactions and assess binding stability and dynamic behaviour using root mean square deviation (RMSD), root mean square fluctuation (RMSF), protein–ligand contacts, radius of gyration (rGyr), intramolecular hydrogen bonds (intraHB), molecular surface area (MolSA), solvent-accessible surface area (SASA), and polar surface area (PSA) [28, 29].

### In vitro experiments

#### Cell culture and treatment

HepG2 (Human hepatocellular carcinoma) and HEK-293 (human non-cancerous cells) cell lines were obtained from the National Centre for Cell Science (NCCS), Pune, India, and were cultured in high-glucose Dulbecco’s Modified Eagle’s Medium (DMEM) supplemented with 10% fetal bovine serum (FBS), 100 U/mL penicillin, and 100 µg/mL streptomycin. Cells were maintained at 37 °C in a humidified atmosphere of 5% CO₂ and subcultured upon reaching 70–80% confluence using 0.25% trypsin-EDTA.

#### MTT cell viability assay

Cell viability was evaluated using the MTT [3-(4,5-dimethylthiazol-2-yl)-2,5-diphenyltetrazolium bromide] assay. Briefly, HepG2 and HEK-293 cells were seeded into 96-well plates at a density of 1 × 10⁴ cells/well and allowed to adhere for 24 h. Cells were then treated with varying concentrations of SP (0–20 mM for HepG2 and 0–80 mM for HEK-293) for 48 h, with cisplatin included as a standard reference control and NaCl (0–20 mM) as an osmotic control. Following the 48-h treatment, 20 µL of MTT solution (5.0 mg/mL in PBS) was added to each well, and the plates were incubated for 4 h at 37 °C. The culture medium was carefully discarded, and the intracellular formazan crystals were dissolved in 100 µL of dimethyl sulfoxide (DMSO) with gentle shaking for 15 min at room temperature. Absorbance was recorded at 570 nm using a microplate reader (Tecan Sunrise). Cell viability was calculated as a percentage relative to the untreated control group. The half-maximal inhibitory concentration (IC₅₀) was determined from dose–response curves using non-linear regression analysis [30]. To evaluate the selective cytotoxicity of SP, the selectivity index (SI) was calculated [31] using the IC₅₀ values of the HEK-293 and HepG2 according to the following equation:

$$\:SI=\frac{{\mathrm{IC}}_{50}\left(\mathrm{HEK-293}\right)}{{\mathrm{IC}}_{50}\left(\mathrm{HepG2}\right)}$$

All experiments were performed in independent triplicates, and the data are presented as mean ± standard deviation (SD).

#### Crystal violet assay

HepG2 cells were seeded in 6-well plates at a density of 2 × 10⁵ cells per well and allowed to adhere for 24 h. Cells were then treated with SP at concentrations of 5 mM, and 10 mM for 48 h at 37 °C in a humidified 5% CO₂ atmosphere. Following treatment, the culture medium was carefully removed, and cells were washed twice with ice-cold PBS to remove non-adherent cells and debris. Adherent cells were fixed with 4% paraformaldehyde for 15–20 min at room temperature, followed by two additional PBS washes. Fixed cells were then stained with 0.1% crystal violet solution (prepare in 20% methanol) for 5 min at room temperature [32]. After staining, wells were rinsed multiple times with distilled water until the wash solution was clear, and plates were air-dried overnight. Crystal violet-stained cells were solubilized by adding 500 µL of Sørensen’s buffer (50% ethanol, 0.1 m sodium citrate) per well, and the optical density was measured at 540 nm using a microplate reader. The absorbance values directly correlate with the number of attached viable cells. Cell viability was expressed as the percentage of stained cells relative to untreated control cells using the formula: (OD of treated cells/OD of control cells) × 100. All experiments were performed in triplicate, and data are presented as mean ± SD. Statistical significance was evaluated using a one-way ANOVA followed by Šidák’s post hoc multiple comparisons test (**P < 0.01, ***P < 0.001).

#### RNA extraction and cDNA synthesis from HepG2 Cells

Total RNA was isolated from cultured HepG2 cells using the TRIzol reagent by manual method. Briefly, cells were washed with cold phosphate-buffered saline (PBS) and lysed by adding TRIzol reagent directly to cells. After phase separation with chloroform and centrifugation, the aqueous phase containing RNA was carefully collected, and RNA was precipitated by isopropanol. The RNA pellet was washed with 75% ethanol, air-dried, and dissolved in RNase-free water. The concentration and purity of the isolated RNA were measured spectrophotometrically by absorbance at 260 nm and 280 nm [33].

For cDNA synthesis, we used the Phi-Script™ 1st Strand cDNA Master mix (5X) (CatLog No. R6203/R6204, DXBIDT Enzyme, Bangalore, India) according to the manufacturer’s instructions. Briefly, 1 µg of RNA was used as template in a 20 µL reaction volume containing 4 µL of 5X cDNA Master mix, 1 µL Phi Script Reverse Transcriptase (200 U/µL), and nuclease-free water. The reverse transcription reaction utilized oligo(dT) primers and reverse transcriptase enzyme, incubated at 42 °C for 60 min followed by reverse transcriptase enzyme inactivation by heating at 95 °C for 2 min.

#### Quantitative real-time PCR analysis

Quantitative real-time PCR (qRT-PCR) amplification was performed using the Dx/DT SYBR Green qPCR kit on a real-time PCR system. Gene-specific primers for *HDAC2* and *HDAC6* were designed and their amplification efficiencies validated. *β-actin* was used as an internal control to normalize gene expression.

Each PCR reaction contained 1 µL of synthesized cDNA, 10 µL of Dx/DT SYBR Green master mix, 0.5 µM of each forward and reverse primer, and nuclease-free water to a total volume of 20 µL. Thermal cycling conditions were: initial denaturation at 95 °C for 10 min, followed by 40 cycles of 95 °C for 15 s, annealing at 58–60 °C for 30 s, and extension at 72 °C for 30 s. Specificity of amplification was confirmed by melt curve analysis [34].

The primer sequences used were:

- *HDAC2* (Forward):5’-CACCTCCGATTCCGAGCTTTC − 3’
- *HDAC2* (Reverse): 5’-GAAACGTGGGGGCGATAGTC-3’
- *HDAC6* (Forward): 5’-CCCCTGCTGCCTCTGTTTTA-3’
- *HDAC6* (Reverse): 5’-GGAATTGCCACAAACGCACT-3’
- *β-actin* (Forward): 5’-TCACCCACACTGTGCCCATCTACGA − 3’
- *β-actin* (Reverse): 5’-CAGCGGAACCGCTCATTGCCAATGG-3’

Relative expression of *HDAC2* and *HDAC6* genes was calculated using the comparative Cq (2^−ΔΔCq) method. Cq values were normalized to β-actin (ΔCq), and fold changes relative to control groups were determined (2^−ΔΔCq) [35]. Data are expressed as mean ± SD of three independent experiments. To determine statistical significance, a one-way ANOVA was performed, followed by Šidák’s post hoc multiple comparisons test (*p < 0.05, **p < 0.01, *** *p* < 0.001).

#### Caspase-3/7 activity assay

HepG2 cells were plated in 6-well plates at a density of 2 × 10⁵ cells per well. Following a 24-hour adherence period, the cells were exposed to SP at concentrations of 5 mM and 10 mM for 48 h under standard culture conditions (37 °C, 5% CO₂). Post-treatment apoptosis was evaluated by quantifying caspase-3/7 enzyme activity using a colorimetric method according to the manufacturer’s protocol (Elabscience, E-CK-A383). The results were expressed as the percentage increase in enzyme activity relative to the control group. Data represent the results of three independent experiments; each performed in triplicate. Statistical analyses were conducted via one-way ANOVA and Šidák’s post hoc test (*p < 0.05, **p < 0.01, ***p < 0.001).

## Results and discussion

### Identification of HCC-associated, differentially expressed, and chemically interactive genes

**Fig. 1:**
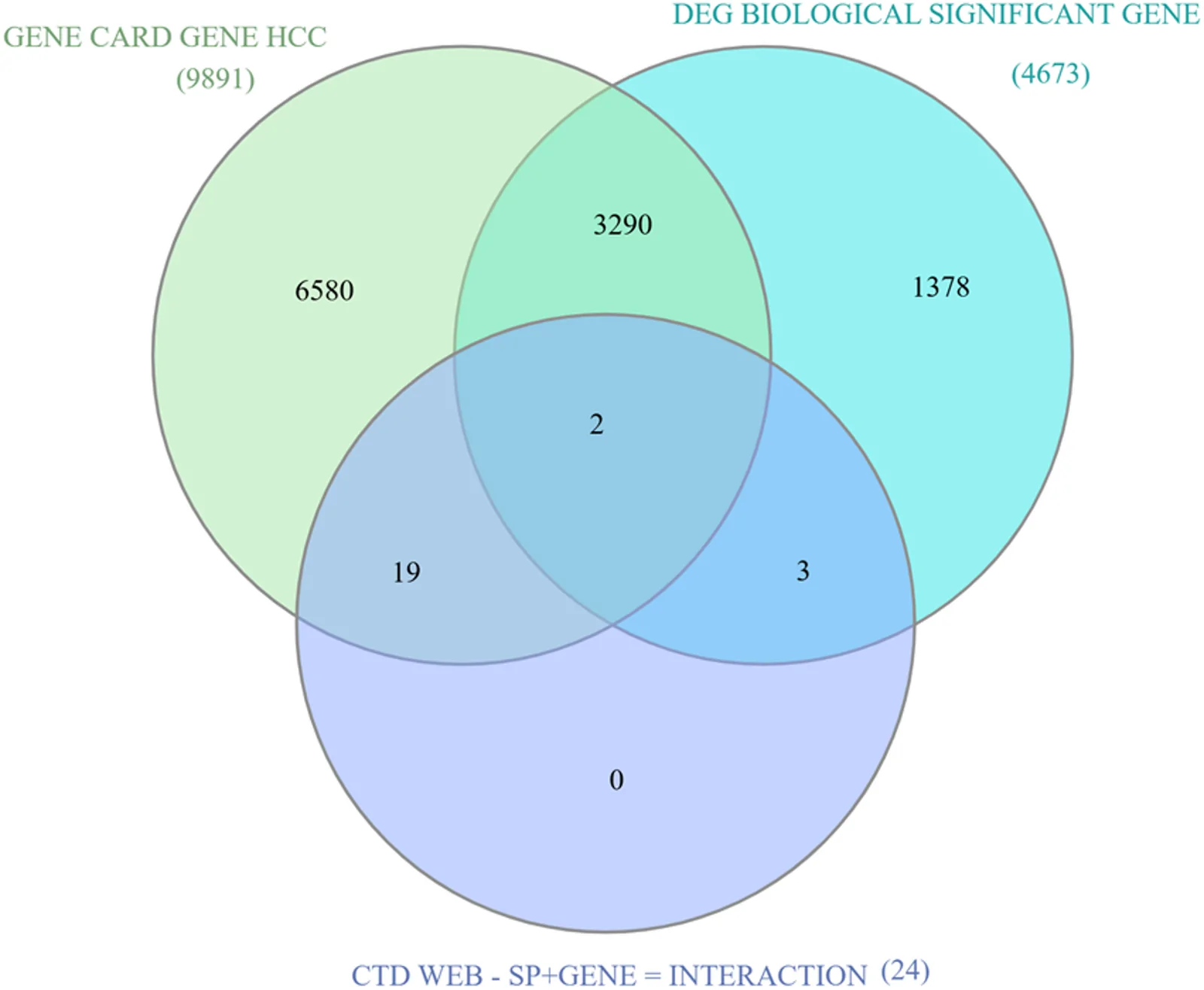
Venn diagram illustrating the overlap between hepatocellular carcinoma (HCC)–associated genes retrieved from GeneCards (*n* = 9,891), differentially expressed genes (DEGs) with significant biological relevance (*n* = 4,673), and chemical–gene interaction targets (*n* = 24)

To identify high-confidence gene targets relevant to hepatocellular carcinoma (HCC), we performed an integrative analysis combining three distinct datasets: (i) HCC-associated genes retrieved from GeneCards (9891 genes), (ii) biologically significant differentially expressed genes (DEGs) identified from transcriptomic profiling (4673 genes), and (iii) chemical–gene interaction data curated from the Comparative Toxicogenomic Database (24 genes). The Venn diagram Fig. 1 analysis revealed a substantial overlap of 3,290 genes between the GeneCards and DEG datasets, indicating good concordance between known hepatocellular carcinoma (HCC)–associated genes and genes that are transcriptionally altered in HCC. This large overlap supports the biological relevance and reliability of the selected gene set. In addition, 6,580 genes were unique to the GeneCards dataset, while 1,378 genes were exclusive to the DEG dataset, reflecting the complementary nature of disease annotation–based and expression-based data sources. Notably, no genes were unique to the chemical–gene interaction dataset, highlighting its highly curated and target-specific nature. Pairwise overlap analysis showed limited intersections involving chemical–gene interactions, with 19 genes shared between GeneCards and chemical–gene interaction data and 3 genes between DEGs and chemical–gene interactions. Importantly, only two genes, *HDAC2* and *HDAC6* were common to all three datasets, representing the most stringently filtered targets supported by disease association, differential expression, and chemical interaction evidence.

Dysregulation of the epigenetic regulators *HDAC2* and *HDAC6* drives HCC progression and therapeutic resistance [36]. Specifically, *HDAC2* promotes tumor proliferation and poor prognosis [6], whereas *HDAC6* regulates non-histone deacetylation, cytoskeletal dynamics, and cancer cell survival [8, 37]. Their consistent identification across multiple datasets highlights these chemically tractable genes as high-confidence therapeutic targets for HCC intervention.

### In silico ADMET profiling of sodium propionate

**Fig. 2:**
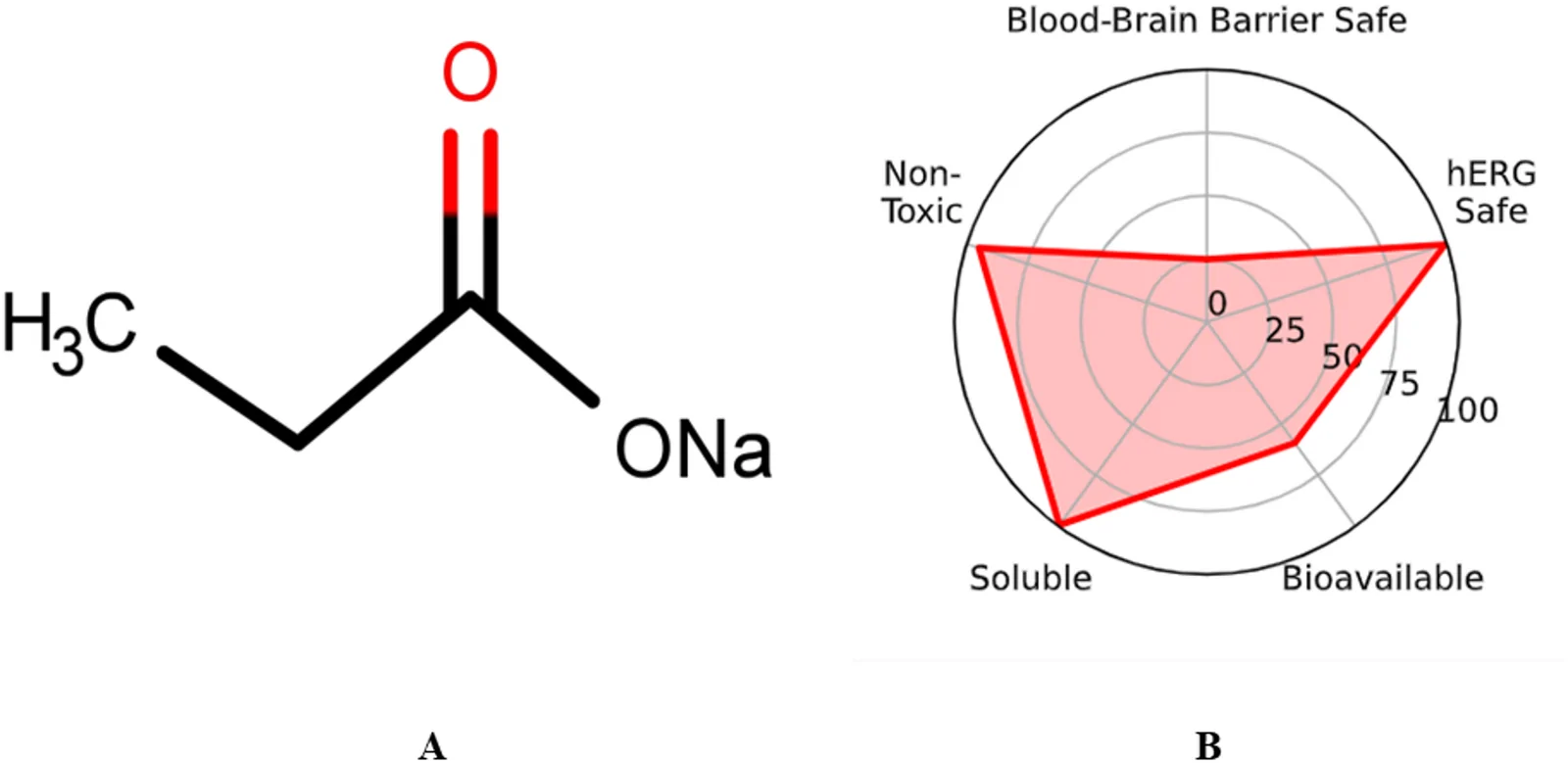
**A** Structure of Sodium propionate (CH3CH2COONa); **B** Radar chart shows high solubility, low toxicity, and favorable blood–brain barrier penetration, with moderate bioavailability and minimal hERG inhibition

In silico ADMET evaluation was conducted to elucidate the drug-likeness, pharmacokinetic behaviour, and safety profile of SP (Fig. 2; Table 1). SP exhibits a low molecular weight (96.06 g/mol), high hydrophilicity (log *P* = − 3.85), and a low topological polar surface area (40.13 Å²). These properties contribute to its complete compliance with Lipinski’s Rule of Five, suggesting excellent oral drug-likeness and suitability for systemic administration [38].

**Table 1:** Predicted ADMET Properties of SP

Property Category	Parameter	Predicted Value	Interpretation
Physicochemical	Molecular Weight (g/mol)	96.06	Low MW
	Log P	-3.85	Highly hydrophilic
	Topological Polar Surface Area (Å²)	40.13	Low
	Lipinski’s Rule of Five	Yes (4/4)	High drug-likeness
Absorption	Human Intestinal Absorption (HIA)	0.899	High
	P-glycoprotein Substrate	0.004	No
	Caco-2 Permeability (log Papp in 10⁻⁶ cm/s)	0.001	Low
Distribution	Blood-Brain Barrier Penetration (BBB)	0.946	Yes
	Volume of Distribution (VDss, L/kg)	0.221	Low
	Plasma Protein Binding (%)	51	Moderate
Metabolism	CYP1A2 Inhibitor	5.95E-05	No
	CYP2C19 Inhibitor	0.0007	No
	CYP2C9 Inhibitor	0.0007	No
	CYP2D6 Inhibitor	8.0E-05	No
	CYP3A4 Inhibitor	2.67E-06	No
Toxicity	Drug-Induced Liver Injury (DILI)	0.417	Low Risk
	hERG Inhibition	0.291	Low Cardiotoxicity Risk
	AMES Mutagenicity	0.036	Non-mutagenic
	Carcinogenicity	0.127	Non-carcinogenic
	Clinical Toxicity	0.003	Low Risk
	Skin Sensitization	0.003	Low Risk

Absorption predictions revealed high human intestinal absorption (HIA = 0.899), suggesting efficient oral uptake Its negligible probability of being a P-glycoprotein substrate suggests minimal efflux-related limitations, which may enhance bioavailability. The low predicted Caco-2 permeability is consistent with its highly hydrophilic and ionizable nature and does not necessarily preclude effective absorption for small molecules [39]. Distribution analysis showed a high probability of blood–brain barrier penetration (BBB = 0.946), suggesting potential central nervous system accessibility.

The low volume of distribution (VDss = 0.221 L/kg) indicates limited tissue accumulation, while moderate plasma protein binding (51%) suggests an adequate free drug fraction for pharmacological activity [40]. Metabolic predictions indicated that SP is not an inhibitor of major cytochrome P450 isoforms (CYP1A2, CYP2C19, CYP2C9, CYP2D6, and CYP3A4), implying a low risk of metabolic liabilities and drug–drug interactions—an important criterion in drug development [41]. Toxicity assessment further supported its safety profile, with a low predicted risk of drug-induced liver injury (DILI = 0.417) and minimal hERG channel inhibition (0.291), indicating low hepatotoxicity and cardiotoxicity risks, respectively. In addition, SP was predicted to be non-mutagenic, non-carcinogenic, and associated with very low clinical toxicity and skin sensitization risks [42]. These results indicate that SP possesses a favourable balance of physicochemical, pharmacokinetic, and toxicological properties, supporting its potential for further therapeutic investigation.

### Molecular docking

Our docking study specifically targeted the orthosteric Zn²⁺-binding catalytic pocket based on its vital role in enzymatic catalysis and established inhibitor recognition. In both Class I (*HDAC2*) and Class IIb *(HDAC6*) enzymes, deacetylation of histone and α-tubulin substrates is strictly dependent on the catalytic Zn²⁺ cofactor and the adjacent transition-state-stabilizing tyrosine residue (TYR308 in *HDAC2*; TYR782 in *HDAC6*) [43, 44]. Well-characterized and FDA-approved *HDAC* inhibitors—including vorinostat, belinostat, panobinostat, and trichostatin A—predominantly bind within this canonical active site by engaging the Zn²⁺ center, occupying the hydrophobic catalytic channel, and interacting with the pocket entrance [44, 45], as validated by co-crystal structures of *N*-(2-aminophenyl) benzamide bound to HDAC2 (PDB: 3MAX) and trichostatin A bound to *HDAC6* (PDB: 5EDU) [44, 46]. Although secondary cavities such as the Class I internal foot pocket and *HDAC6* catalytic domain 1 (CD1) exist [47, 48], primary deacetylase activity resides within the canonical Zn²⁺-dependent active site; therefore, this study focused on evaluating inhibition at the orthosteric catalytic pocket without independent allosteric screening.

**Fig. 3:**
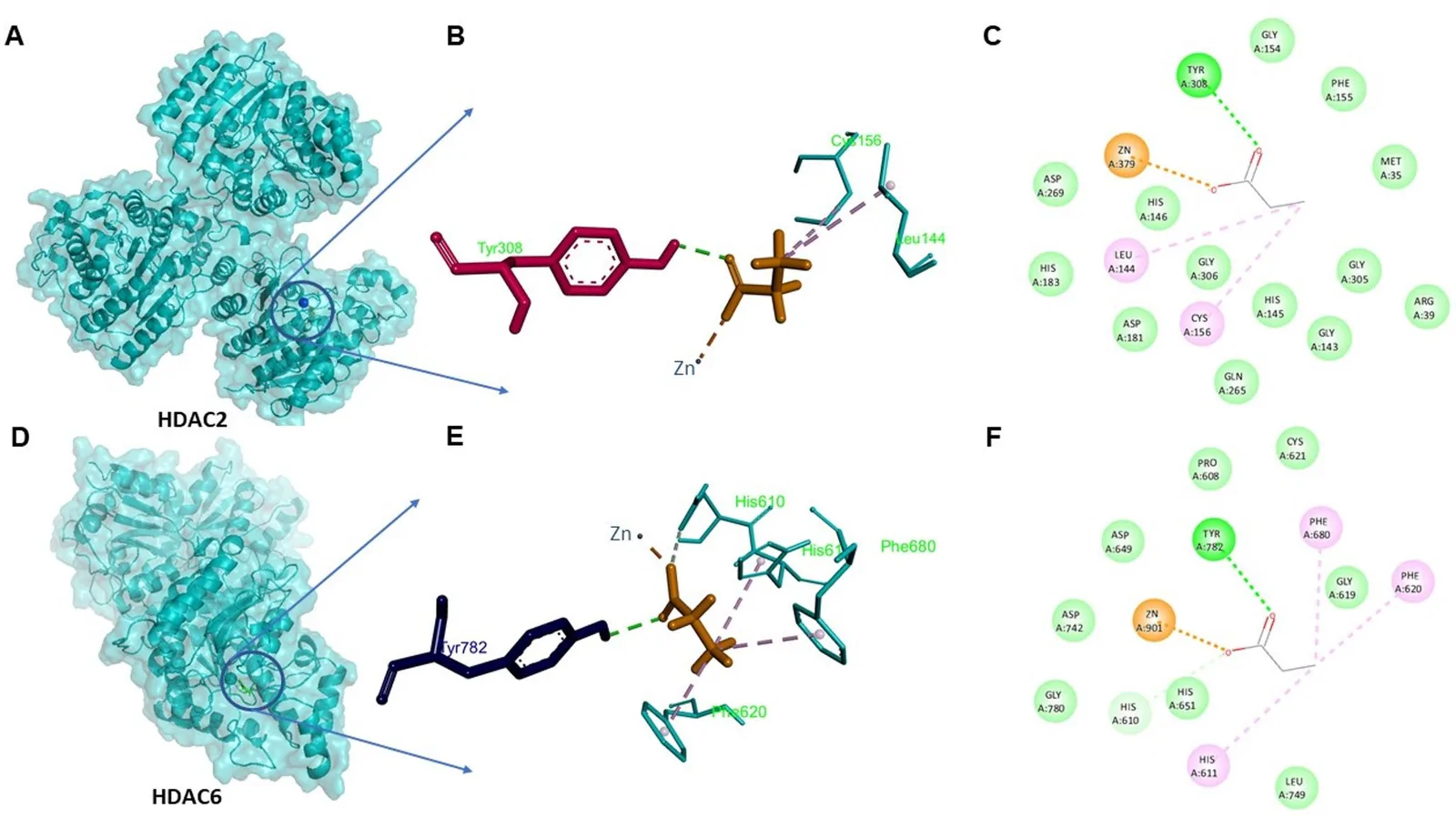
**A**&**D** Depiction of surface and cartoon representation of the *HDAC2 & HDAC6* protein targets with binding sites. **B**&**E**: Highlights the interacting residues of the protein targets with binding residues and Zn²⁺ ion **C**&**F**: representation of the 2D interactions of the protein-ligand complex with hydrogen, alkyl, pi-Alkyl bonds with hydrophobic interactions

Prior to evaluating our compound, the docking protocol was validated by re-docking the co-crystallized ligands (*N*-(2-aminophenyl) benzamide for *HDAC2*; trichostatin A for *HDAC6)* within a grid centered on the Zn²⁺ ion. The optimal docked poses superimposed onto their native experimental structures with an RMSD of ≤ 1.0 Å, validating the chosen docking parameters. Subsequent molecular docking analysis indicated that SP could occupy the catalytic active-site regions of both *HDAC2* and *HDAC6*, showing predicted binding energies of − 5.7 kcal/mol and − 5.6 kcal/mol, respectively (Fig. 3A and D). Notably, SP was positioned in close proximity to the catalytic Zn²⁺ ion, a key structural feature of Zn²⁺-targeting *HDAC* inhibitors, including next-generation inhibitor designs [49–51]. The predicted Zn²⁺–ligand interactions showed favourable short interaction distances (Fig. 3B and E), together with additional hydrogen-bonding and hydrophobic interactions within the active-site pockets (Fig. 3C and F).

Comparing the docked poses of SP with known inhibitors reveals that SP retains the core pharmacophoric interactions of classical *HDAC* inhibitors [46, 52]. Similar to the zinc-binding groups of *N*-(2-aminophenyl) benzamide, trichostatin A, and vorinostat [43, 44, 46], the polar moiety of SP engages in a key electrostatic/metal-coordination interaction with the catalytic Zn²⁺ cofactor (Zn379 in *HDAC2*; Zn901 in *HDAC6*) within the conserved “Asp-His-Asp” pocket (Asp181/His145/His146/Asp269 in *HDAC2*; Asp649/His610/His651/Asp742 in *HDAC6*) (Fig. 3B–C and E–F). Furthermore, SP forms a direct hydrogen bond with the conserved catalytic tyrosine—TYR308 in HDAC2 and TYR782 in *HDAC6* (Fig. 3B–C and E–F). In crystallographic complexes of *HDAC2* (PDB: 3MAX, 4LXZ) and *HDAC6* (PDB: 5EDU, 5EEI), hydrogen bonding with TYR308 and TYR782 is essential for mimicking the transition state during deacetylation inhibition [43, 44, 46]. Additionally, the non-polar tail of SP mimics the linker region of established inhibitors by forming alkyl and π-alkyl interactions with Leu144 and Cys156 in the *HDAC2* pocket (Fig. 3C), and engaging the hydrophobic tunnel residues Phe620, Phe680, and His611 in *HDAC6* (Fig. 3F), which typically stabilize the linkers of trichostatin A, belinostat, and panobinostat [44, 53].

The comparable docking scores and conserved Zn²⁺-proximal binding mode suggest that SP has the potential to interact with both *HDAC2* and *HDAC6.* Unlike clinical inhibitors (− 8 to − 10 kcal/mol) [51, 54, 55], SP lacks a bulky aromatic surface-capping group, accounting for its more modest binding affinities (− 5.7 and − 5.6 kcal/mol) while preserving essential active-site interactions. Because these observed interactions provided a promising structural basis for further investigation, the docked complexes were subsequently subjected to molecular dynamics simulations to assess the stability and persistence of the predicted protein–ligand interactions.

### Molecular dynamics

**Fig. 4:**
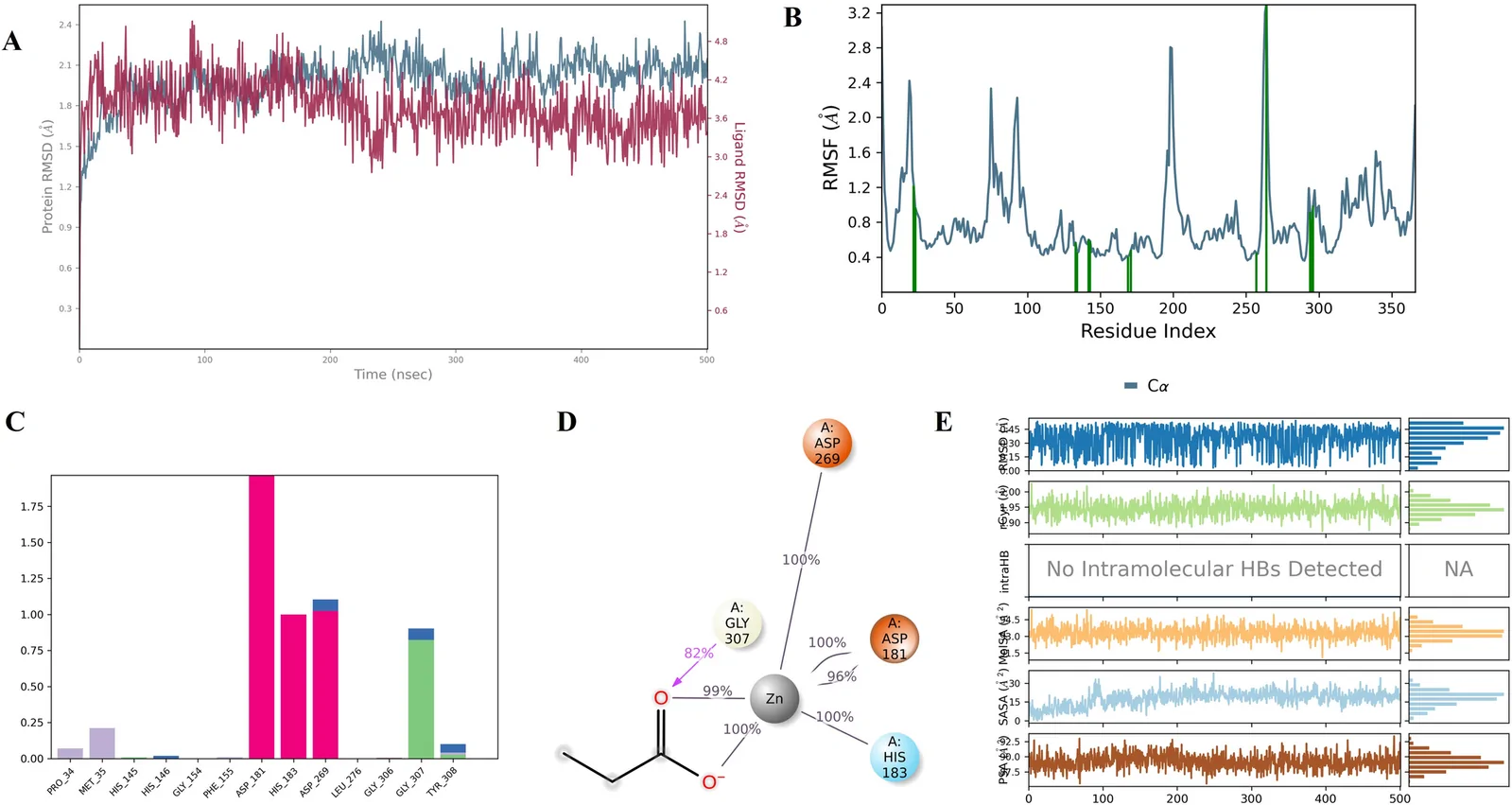
Molecular dynamics simulation analysis of the *HDAC2*-Propionate complex: **A** RMSD depicting structural stability over the simulation period of 500ns; **B** RMSF illustrating residue-wise flexibility of the protein backbone; **C** Protein-ligand interaction profile showing the frequency; **D** Protein-ligand interaction profile showing the types of intermolecular contacts throughout the trajectory of 500ns; **E** Ligand property analysis showing variations in RMSD, radius of gyration (rGyr), MolSA, SASA, and PSA during the simulation

The molecular dynamics (MD) trajectory analysis of the *HDAC2*–Propionate complex provided detailed insights into the structural stability and interaction dynamics of the system. The RMSD profile (Fig. 4A) demonstrated that, following the initial equilibration phase, the *HDAC2* backbone attained structural stability with RMSD values maintained around 3.0-3.5 Å throughout the 500 ns simulation period. In contrast, the ligand RMSD exhibited fluctuations between 1.6 and 2.0 Å during the initial 0-200 ns interval and subsequently between 2.0 and 2.2 Å during the 200–500 ns period. Additionally, another phase of ligand fluctuation was observed in the range of 3.6–4.8 Å during the first 200 ns, followed by stabilization between 3.0 and 4.0 Å thereafter. These fluctuations suggest minor conformational rearrangements of the ligand within the binding pocket while preserving stable protein-ligand interactions. Overall, the results confirm the stability of the docked conformation and the persistence of favourable *HDAC2*-Propionate interactions throughout the simulation trajectory. The RMSF analysis revealed the most residues fluctuated below 0.8Å, including active site residues 33–35, 144–146, 154–156, 181, and 305–308, indicating overall structural stability of the *HDAC2*–propionate complex during the simulation, with minor fluctuations only minor fluctuations observed at residues 276, 211, 105, 87, 30 reaching upto 3.0 Å (Fig. 4B).

The protein-ligand interaction histogram (Fig. 4C) demonstrated that hydrogen bonding and ionic bond interactions predominated, particularly hydrogen bonds with GLY307 and TYR308, along with ionic interactions involving ASP181, HIS183, and ASP269, these interaction were further validated by the ligand-protein contact map (Fig. 4D), which showed persistent hydrogen bonding with GLY307 (82% occupancy), and stable Zinc mediated metal coordination involving the carbonyl oxygen and anionic oxygen of the propionate carboxylate group, exhibiting occupancies of 99% and 100%, respectively, throughout the 500 ns simulation. Furthermore, ligand property analyses indicated stable behaviour across the trajectory, with minimal fluctuations in RMSD (-0.01-0.5 Å), rGyr (1.85-2.0 Å), MolSA (91–95 Å^2^), SASA (10–30 Å^2^), PSA (87–93 Å^2^). Collectively, these findings suggest that propionate forms a dynamically stable complex with *HDAC2* through a combination of persistent hydrogen bonding, ionic interactions, and strong Zn²⁺ metal coordination, thereby contributing to the overall conformational stability of the complex.

The ligand Propionate in our study exhibited coordination interaction with the catalytic Zn²⁺ ion within the *HDAC* active site, suggesting its potential role as a zinc-chelating Histone Deacetylase Inhibition inhibitor capable of suppressing enzymatic activity. The zinc-binding group (ZBG) of *HDAC* inhibitors is crucial for coordinating with the catalytic Zn²⁺ ion and thereby blocking deacetylase function. Consequently, next-generation *HDAC* inhibitors research is largely focused on designing optimized ZBGs with improved zinc-chelation, enhanced isoform selectivity, reduced toxicity, and superior pharmacokinetic properties for safer and more effective anticancer therapy [49–51]. This high-affinity zinc chelation mechanism is also responsible for the therapeutic efficacy of clinically important *HDAC* inhibitors such as Vorinostat, Belinostat, and Panobinostat.

Molecular docking and MD simulations provide complementary insights into the possible binding mode, key molecular interactions, and stability of protein-ligand complexes. While additional analyses such as principal component analysis (PCA), free-energy landscape (FEL) analysis, and characterization of representative low-energy conformations can provide a more detailed understanding of protein motions and conformational states [56, 57]. The computational studies in the present study were limited to molecular docking and MD simulations; these approaches were employed to obtain preliminary structural insights into the interaction of SP with *HDAC2* and *HDAC6* and to assess the stability and persistence of the predicted complexes. The computational findings were then taken forward for experimental evaluation through HepG2 cytotoxicity assays, qRT-PCR analysis of *HDAC2* and *HDAC6* expression and caspase 3/7 activity analysis. This combined approach allowed the computational predictions to be examined in a biological context and provided complementary evidence for the potential of SP to modulate *HDAC2* and *HDAC6*.

### Effect of sodium propionate on cell viability

**Fig. 5:**
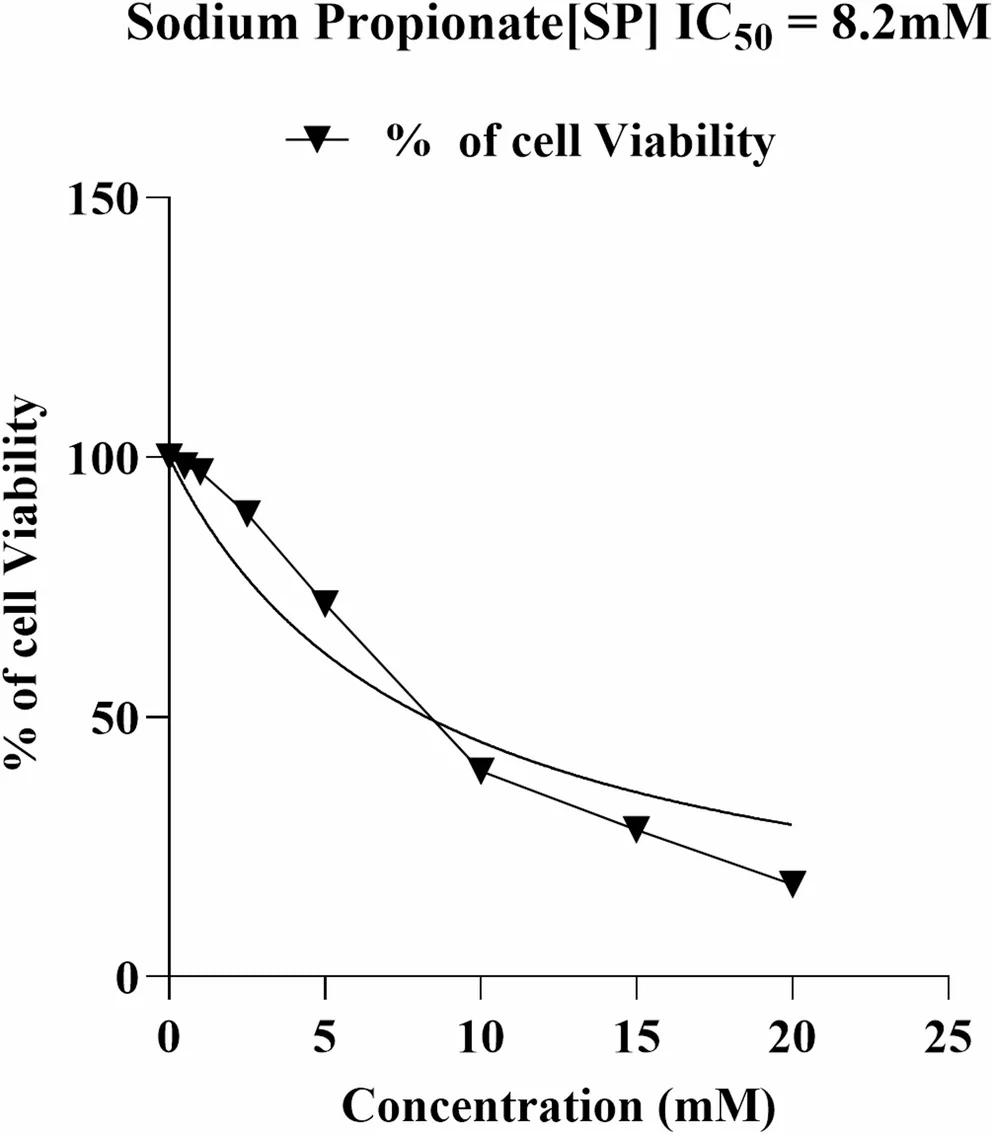
Cytotoxicity of SP in HepG2 cell line. Dose–response curve of cells treated with SP for 48 h. Viability was measured via MTT assay (*n* = 3). The calculated IC50 was 8.2 mM

The cytotoxic potential of SP against HepG2 cells was evaluated across a concentration range of 0–20 mM, revealing a distinct dose-dependent reduction in cell viability (Fig. 5). Initial observations indicated that lower concentrations of SP (up to 2.5 mM) were well-tolerated by the cells, maintaining viability at approximately 100% relative to the control. However, a sharp decline in cell viability was observed between 5 mM and 10 mM, yielding an IC₅₀ value of 8.2 mM and demonstrating notable cytotoxicity against the HepG2 cancer cell line. The standard reference drug, cisplatin, exhibited an IC₅₀ of 11.35 µM under the same conditions. Importantly, the osmotic control (NaCl) induced no observable cytotoxicity at equivalent tested concentrations. This confirms that the anti-proliferative activity observed at millimolar concentrations of SP is a targeted pharmacological effect, rather than a non-specific consequence of ionic or osmotic stress.

To assess the specific targeted action of SP, a selectivity index (SI) was calculated. While SP demonstrated significant toxicity toward HepG2 cells, it exhibited a much higher tolerance in human non-cancerous HEK-293 cells, with an IC₅₀ of 29.12 mM. This resulted in an SI value of 3.51, indicating a favourable therapeutic window and demonstrating that SP possesses strong selective cytotoxicity toward cancer cells over normal healthy cells.

Consistent with these findings, previous studies demonstrate that SP induces dose-dependent cytotoxicity across different cancer models, though with highly variable, cell-type-dependent IC50 values. Specifically, while concentrations of 10–30 mM significantly reduce viability in breast cancer cells (MCF-7, SK-BR-3, and MDA-MB-231) [58], ovarian cancer cell lines SKOV-3 and PA-1 demonstrate IC50 values of 15.7 mM and 8.8 mM at 72 h, respectively [59]. Glioblastoma models can exhibit even lower sensitivity, with the A172 cell line requiring an IC50 of 41.21 mM at 72 h to achieve similar cytotoxic effects [60].

### Crystal violet assay

**Fig. 6:**
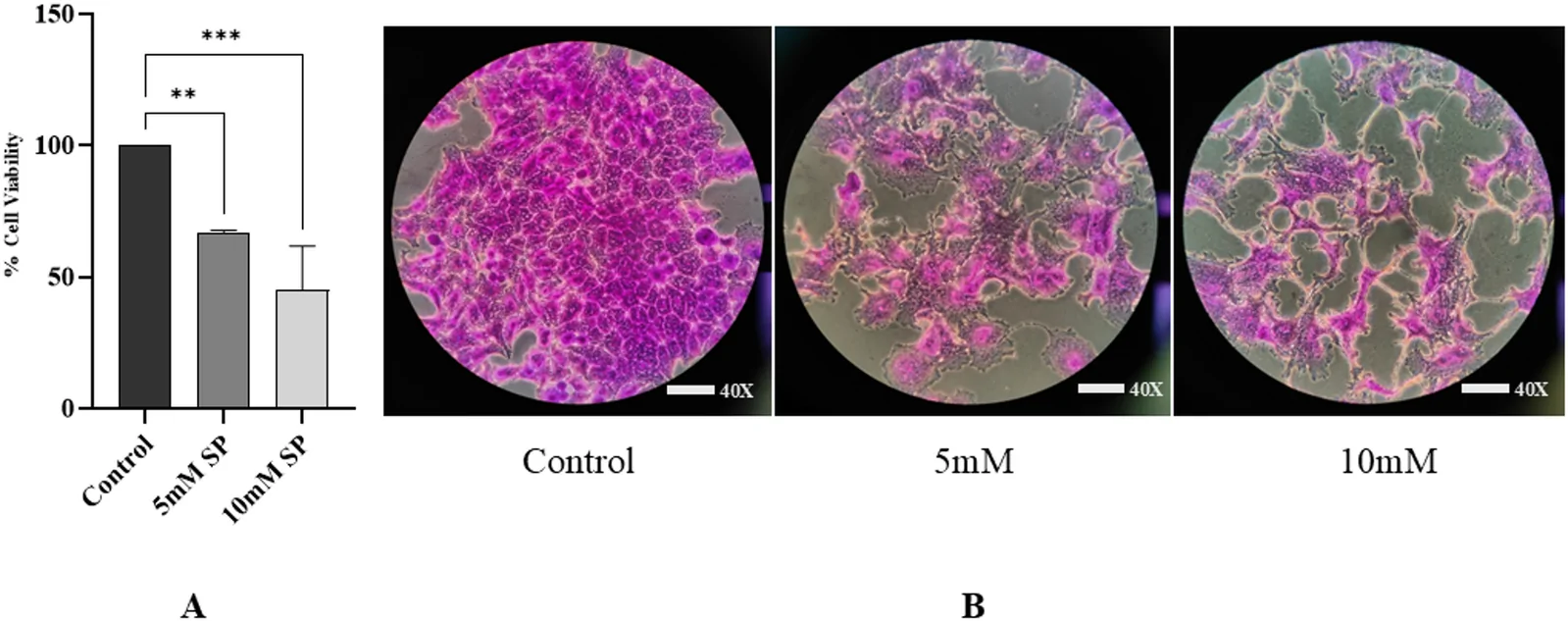
Crystal violet staining. **A** Bar graph representing the effect of SP on HepG2 cell viability assessed by crystal violet assay. **B** Microscopic images (40× magnification) of crystal violet-stained cells after treatment with 5 mM and 10 mM SP (Significance level: *p < 0.05, **p < 0.01, *** *p* < 0.001)

The anti-proliferative activity of SP against HepG2 hepatocellular carcinoma cells was validated using the crystal violet staining assay, which assesses cell viability and morphological integrity. As shown in Fig. 6A, untreated HepG2 cells exhibited 100% viability, reflecting normal cell growth and adherence. In contrast, treatment with SP resulted in a significant and concentration-dependent reduction in cell viability. At 5 mM concentration, cell viability decreased to approximately 69.01%, representing a statistically significant reduction compared to the control group (*p* < 0.05). A further increase in concentration to 10 mM led to a more pronounced decrease in viability to approximately 48.91%, which was highly significant when compared to both control and 5 mM-treated groups (*p* < 0.01). These findings corroborate the cytotoxic effects observed in the MTT assay, confirming the anti-proliferative potential of SP against HepG2 cells. The microscopic observations (40X magnification) provide visual confirmation of these results Fig. 6B. While the control group shows a confluent and densely packed monolayer of cells with typical morphology, cells treated with 5 mM and 10 mM of SP exhibit a visible reduction in cell number and an increase in intercellular spacing. Morphological alterations, including cell shrinkage and loss of characteristic shape.

### Sodium propionate suppresses *HDAC2* and *HDAC6* expression in HepG2 cells

**Fig. 7:**
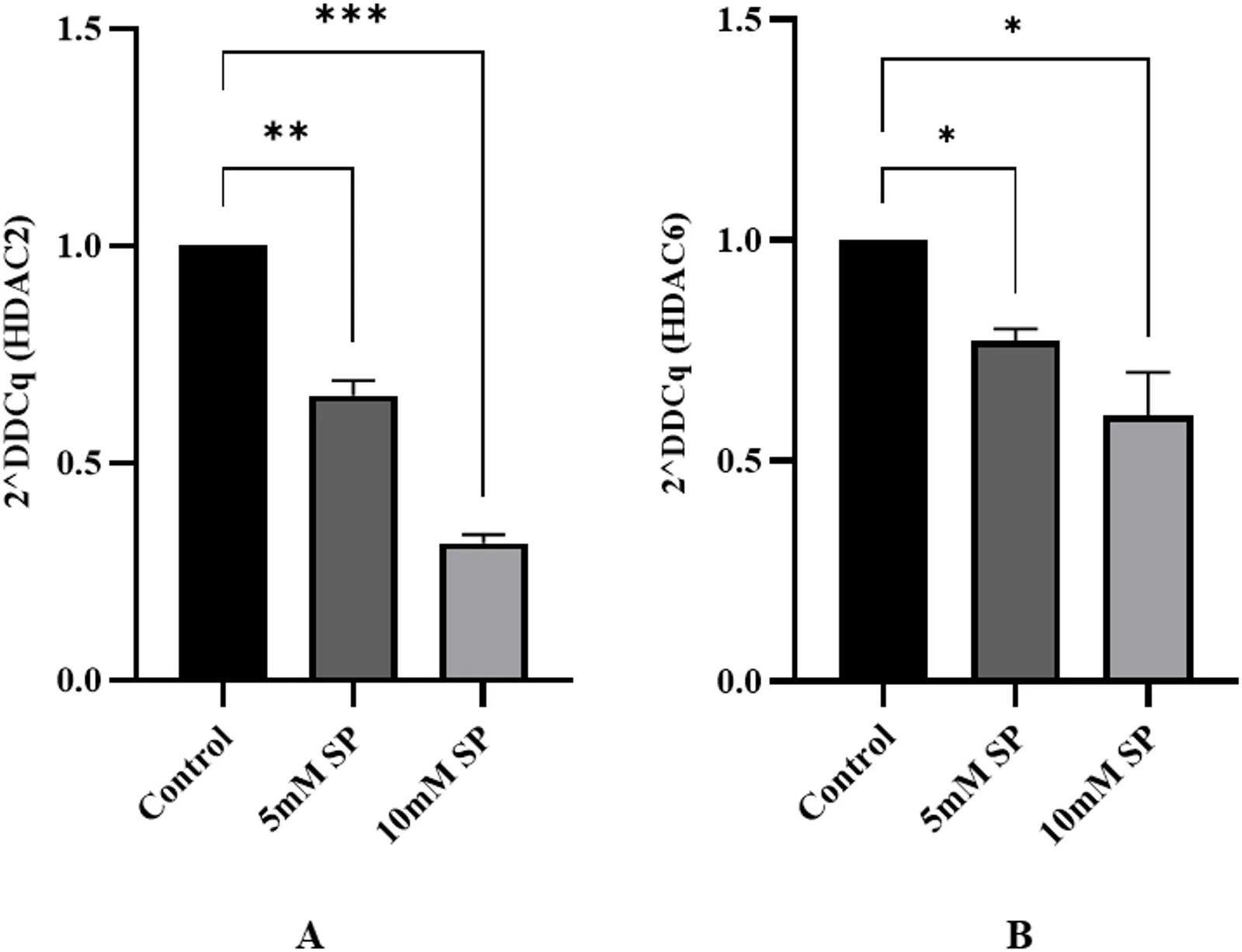
qRT-PCR analysis. **A***HDAC2* and **B***HDAC6* expression levels in control and SP-treated cells. (*p < 0.05, **p < 0.01, *** *p* < 0.001)

Quantitative real-time PCR analysis revealed a significant and dose-dependent downregulation of *HDAC2* expression in SP treated HepG2 cells compared to control Fig. 7A. Treatment with 5 mM SP reduced *HDAC2* mRNA levels to 0.65-fold, while 10 mM SP further suppressed expression to 0.31-fold, with reductions statistically significant (**P < 0.01 and ***P < 0.001).

*HDAC2* overexpression in HCC drives tumor progression and poor prognosis [6, 36] by repressing tumor suppressors and activating the mTORC1/AKT proliferative axis [61]. By suppressing *HDAC2*, SP restores acetylation balance and inhibits oncogenic transcription, mirroring how short-chain fatty acids act as HDAC inhibitors to induce apoptosis and cell cycle arrest [62].

Similarly, *HDAC6* expression was also significantly reduced in SP-treated HepG2 cells Fig. 7B. Treatment with 5 mM and 10 mM SP decreased *HDAC6* expression to approximately 0.77-fold and 0.60-fold, respectively (*P < 0.05). *HDAC6* drives oncogenic signaling and stress responses by deacetylating non-histone substrates like α-tubulin and HSP90 [8, 61, 63]. Its overexpression in HCC promotes tumor growth, metastasis, and therapy resistance [37, 64, 65]; therefore, the downregulation of *HDAC6* by SP suggests its anticancer effects stem from disrupting these critical cytoplasmic survival pathways.

### Caspase-3/7 activity assay

**Fig. 8:**
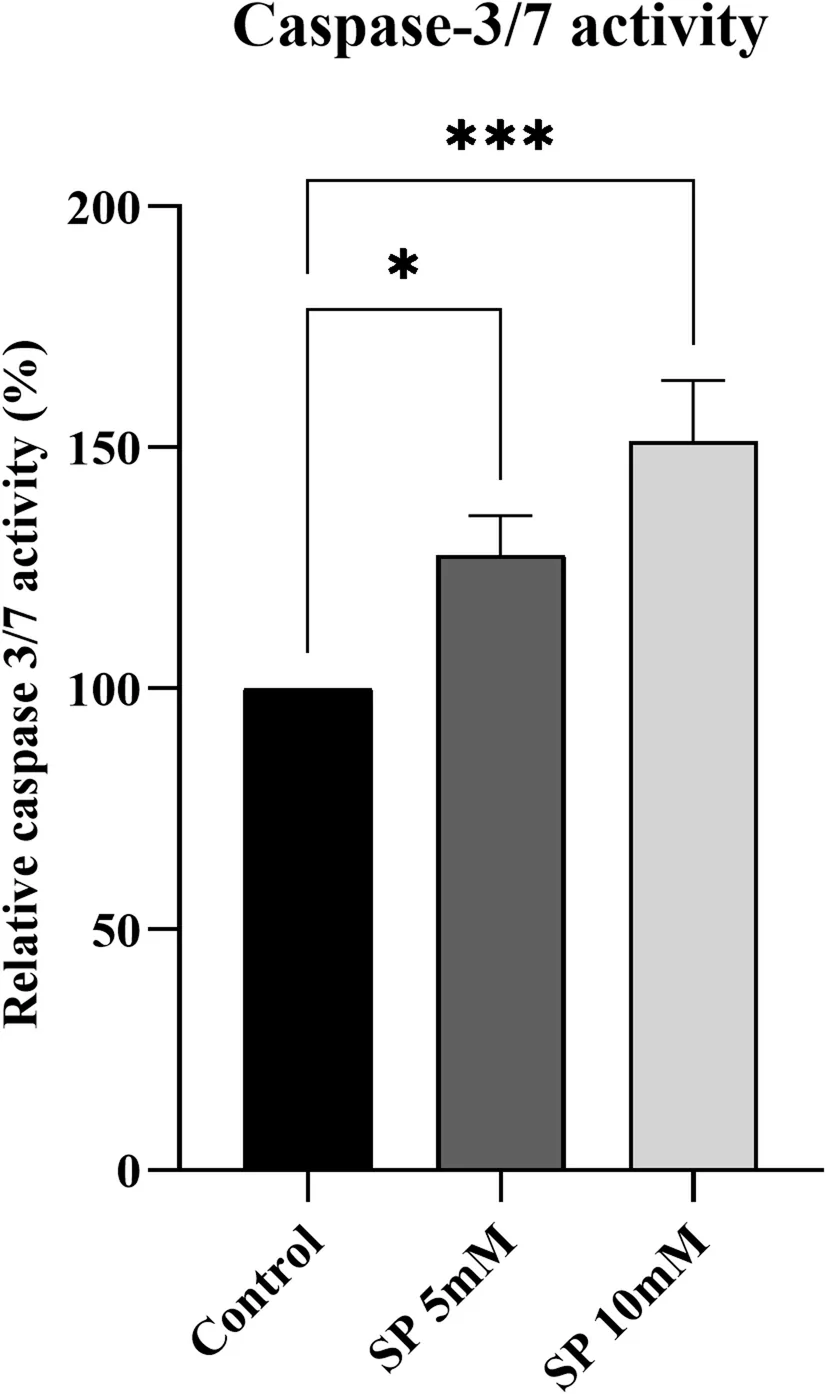
Caspase-3/7 activity in SP-treated HepG2 cells (*n* = 3). (* *p* < 0.05, ** *p* < 0.01, *** *p* < 0.001)

To investigate the functional effects of SP-induced cytotoxicity, caspase-3/7 activity was evaluated in cells treated with 5 mM and 10 mM SP (Fig. 8). The results demonstrated a significant, dose-dependent increase in caspase-3/7 activity. Compared with the untreated control, cells treated with 5 mM SP showed a significant increase in caspase-3/7 activity (*p* < 0.05), which was further increased at 10 mM SP (*p* < 0.001), indicating activation of caspase-dependent apoptosis.

SP acts as a dual-action epigenetic modulator with inhibitory effects against HCC cells. In silico molecular docking suggests that SP may interact directly with the catalytic sites of *HDAC2* and *HDAC6*, with binding affinities of − 5.7 and − 5.6 kcal/mol, respectively. The *HDAC2*–SP complex is stabilized by persistent hydrogen bonds with TYR308 and GLY307 and coordination with the catalytic Zn²⁺ ion, suggesting a possible zinc-chelating interaction similar to that of established *HDAC* inhibitors. Similarly, SP forms stable interactions with TYR782 in *HDAC6*. Moreover, 500 ns molecular dynamics simulations supported the stability of both protein–ligand complexes, with RMSD values of 3.0–3.5 Å and active-site RMSF values below 0.8 Å.

In addition to this predicted enzymatic inhibition, SP also suppresses these targets at the transcriptional level. In HepG2 cells, SP treatment produced a dose-dependent reduction in *HDAC2* and *HDAC6* mRNA expression. This dual suppression was directly associated with the activation of caspase-dependent apoptosis. At a 5 mM concentration of SP, the reduction in *HDAC* expression was associated with a significant increase in caspase-3/7 activity (*p* < 0.05). At 10 mM, further suppression of both *HDAC* transcripts was accompanied by a much greater increase in caspase-3/7 activity (*p* < 0.001). This parallel dose-dependent response strongly links SP-induced *HDAC* modulation with the activation of apoptotic signalling. This observation is consistent with existing literature showing that *HDA*C dysregulation promotes HCC progression and that its inhibition can promote cell death. In HCC, *HDAC* inhibition has been shown to activate the apoptosome by increasing apoptotic protease-activating factor-1 (Apaf-1), thereby promoting caspase-dependent apoptosis [66]. In addition, modulation of the *HDAC*/survivin axis has been directly associated with enhanced apoptotic responses and caspase-3 activation in HepG2 cells [67].

These findings suggest that SP acts as a dual-action epigenetic modulator by reducing *HDAC2* and *HDAC6* mRNA expression while potentially interfering with their direct enzymatic activity. Together, these mechanisms may contribute to the apoptotic activity observed in HCC cells. While this study is limited by the absence of direct enzymatic assays and protein-level validation, these findings provide a basis for future experimental work to confirm the predicted inhibition and map the precise molecular pathways.

## Conclusion

This study identifies sodium propionate (SP) as a potential epigenetic modulator of hepatocellular carcinoma (HCC) through its effects on *HDAC2* and *HDAC6*. Integrative bioinformatics identified *HDAC2* and *HDAC6* as common targets, while molecular docking and molecular dynamics simulations indicated stable interactions of SP within their catalytic sites, including coordination with the catalytic Zn²⁺ ion. In HepG2 cells, SP reduced cell viability in a concentration-dependent manner (IC₅₀ = 8.2 mM), suppressed *HDAC2* and *HDAC6* mRNA expression, and increased caspase-3/7 activity, supporting an association between *HDAC* modulation and apoptotic cell death. ADMET analysis further indicated favorable drug-likeness and high predicted intestinal absorption, although the high hydrophilicity and low Caco-2 permeability may represent barriers to efficient cellular and systemic exposure. These findings provide integrated computational and experimental evidence for the anticancer potential of SP and support further investigation of SP–*HDAC* interactions in HCC. However, direct biochemical assays are required to validate the predicted enzymatic inhibition, along with protein-level confirmation and comprehensive evaluation in in vivo HCC models.

## Data Availability

No datasets were generated or analysed during the current study.
